# Computational microbiome pharmacology analysis elucidates the anti-cancer potential of vaginal microbes and metabolites

**DOI:** 10.3389/fmicb.2025.1602217

**Published:** 2025-09-16

**Authors:** Damilola C. Lawore, Smrutiti Jena, Alicia R. Berard, Kenzie Birse, Alana Lamont, Romel D. Mackelprang, Laura Noel-Romas, Michelle Perner, Xuanlin Hou, Elizabeth Irungu, Nelly Mugo, Samantha Knodel, Timothy R. Muwonge, Elly Katabira, Sean M. Hughes, Claire Levy, Fernanda L. Calienes, Florian Hladik, Jairam R. Lingappa, Adam D. Burgener, Leopold N. Green, Douglas K. Brubaker

**Affiliations:** ^1^Weldon School of Biomedical Engineering, Purdue University, West Lafayette, IN, United States; ^2^Department of Obstetrics and Gynecology, University of Manitoba, Winnipeg, MB, Canada; ^3^Center for Global Health and Diseases, Department of Pathology, Case Western Reserve University School of Medicine, Cleveland, OH, United States; ^4^Department of Global Health, University of Washington, Seattle, WA, United States; ^5^Department of Medical Microbiology and Infectious Disease, University of Manitoba, Winnipeg, MB, Canada; ^6^Partners in Health Research and Development, Kenya Medical Research Institute, Nairobi, Kenya; ^7^Infectious Diseases Institute, Makerere University, Kampala, Uganda; ^8^Department of Obstetrics and Gynecology, University of Washington, Seattle, WA, United States; ^9^Fred Hutchinson Cancer Research Center, Seattle, WA, United States; ^10^Department of Medicine, University of Washington, Seattle, WA, United States; ^11^Department of Pediatrics, University of Washington, Seattle, WA, United States; ^12^Department of Medicine Solna, Karolinska Institutet, Solna, Sweden; ^13^The Blood Heart Lung Immunology Research Center, Case Western Reserve University, University Hospitals of Cleveland, Cleveland, OH, United States

**Keywords:** vaginal microbial community, gynecological cancer, anti-cancer, systems biology, multi-omics

## Abstract

The vaginal microbiome's role in risk, progression, and treatment of female cancers has been widely explored. Yet, there remains a need to develop methods to understand the interaction of microbiome factors with host cells and to characterize their potential therapeutic functions. To address this challenge, we developed a systems biology framework we term the Pharmacobiome for microbiome pharmacology analysis. The Pharmacobiome framework evaluates similarities between microbes, microbial byproducts, and known drugs based on their impact on host transcriptomic cellular signatures. Here, we apply our framework to characterization of the Anti-Gynecologic Cancer Vaginal Pharmacobiome. Using published vaginal microbiome multi-omics data from the Partners PrEP clinical trial, we constructed vaginal epithelial gene signatures associated with each profiled vaginal microbe and metabolite. We compared these microbiome-associated host gene signatures to post-drug perturbation host gene signatures related to 35 FDA-approved anti-cancer drugs from the Library of Integrated Network-based Cellular Signatures database to identify vaginal microbes and metabolites with high statistical and functional similarity to these drugs. We found that select lactobacilli particularly *L. crispatus* and their metabolites, such as taurine, can regulate host gene expression in ways similar to certain anti-cancer drugs. Additionally, we experimentally tested our model prediction that taurine, a metabolite produced by *L. crispatus*, kills cancerous breast and endometrial cancer cells. Our study shows that the Pharmacobiome is a robust framework for characterizing the anti-cancer therapeutic potential of vaginal microbiome factors with generalizability to other cancers, microbiomes, and diseases.

## Introduction

The vaginal microbiota consists of a diverse range of beneficial microbes and potential pathogens that reside within the vaginal environment (Smith and Ravel, 2017; Gajer et al., 2012). Techniques like metagenomics, metatranscriptomics, metaproteomics, and metabolomics have laid the foundation for understanding the complexity of the vaginal microbiome and highlighted the importance of specific bacteria spp., like *Lactobacillus* sp. in maintaining a healthy vaginal environment (Chen et al., 2021; Barrientos-Durán et al., 2020). The vaginal microbiota varies during a woman's life and the menstrual cycle, making dysbiosis of the vaginal microbiota challenging to diagnose. Nonetheless, vaginal microbiota lacking *Lactobacillus* spp. have been associated with various disease states, including persistent HPV infection, an increased risk of sexually transmitted infections (STI), pelvic inflammatory disease, preterm birth, poor obstetric outcomes, infertility, and the development of gynecologic cancers, particularly cervical cancer. Emerging evidence suggests potential links to endometrial and ovarian cancers, although further research is needed to confirm these associations (Martin and Marrazzo, 2016; Lewis et al., 2017; Nunn and Forney, 2016; Lev-Sagie et al., 2022; Holdcroft et al., 2023; Cheng et al., 2020).

The vaginal microbiome has been the subject of increasing interest in the context of gynecological cancers, where studies have shown that dysbiosis may induce epithelial barrier dysfunction, immunological dysregulation, genotoxicity, and inflammation, resulting in a tumor-permissive microenvironment (Rajagopala et al., 2017; Garrett, 2015). Bacteria, like *Chlamydia trachomatis*, can trigger epithelial-to-mesenchymal transition (EMT) in host epithelial cells (Caven and Carabeo, 2023; Zadora et al., 2019). This process involves transforming epithelial cells into a mesenchymal phenotype, which can lead to decreased cell adhesion and increased motility. Such changes are significant as they may contribute to tissue fibrosis and potentially to carcinogenesis (Zadora et al., 2019).

Additionally, several studies have noted an association between the depletion of *Lactobacillus* spp. and increased diversity in the vaginal microbiota in women with gynecologic cancers, including cervical and endometrial cancer. However, the association with ovarian cancer is less established (Ventolini et al., 2022; Javadi et al., 2024). Cervical cancer is strongly linked to persistent infection with high-risk human papillomavirus (HR-HPV) types, While certain bacterial spp. such as *Sneathia sanguinegens, Anaerococcus tetradius*, and *Peptostreptococcus anaerobius* have been studied in relation to HPV infection and cervical carcinogenesis, but the evidence is not yet conclusive regarding their roles (Mitra et al., 2015). Research into the role of specific bacterial spp. in endometrial and ovarian cancers is ongoing. Some studies have identified associations between certain bacteria and these cancers, but the findings are not definitive. For instance, *Porphyromonas* spp. has been noted in endometrial cancer patients. However, more comprehensive studies are required to confirm these associations and understand the underlying mechanisms (Hakimjavadi et al., 2022).

Furthermore, bacterial communities can potentially play a role in the development, severity, and treatment response of gynecological malignancies. Due to sparse information on modulatory effects, the intricate relationship between the vaginal microbiota and gynecological cancers is not fully elucidated (Chase et al., 2015). One key challenge is the heterogeneity observed in vaginal microbiomes across individuals. The diversity and abundance of microbial spp. can vary significantly among women due to age, ethnicity, hormonal status, sexual practices, antibiotic use, and hygiene habits (Ravel et al., 2011). This inherent variability makes it challenging to establish a standard reference or define a healthy vaginal microbiome profile applicable to all women. While evidence suggests that alterations in the vaginal microbiota may contribute to an increased risk of certain cancers (e.g., cervical or endometrial cancer), the exact mechanisms behind this interplay remain unclear (Walther-António et al., 2016).

The vaginal microbiome also plays a significant role in influencing the response to cancer therapy by regulating inflammation in the context of gynecologic cancers (Łaniewski et al., 2020; Ventolini et al., 2022; Kudela et al., 2023). Additionally, intraperitoneal bacterial infections have been linked to adverse events in ovarian cancer patients (Manyam et al., 2021). It is essential to recognize that the disruption of the vaginal microbiome can have indirect consequences on the efficacy of pelvic cancer treatments and the healing process following surgery (Kudela et al., 2023; Kyrgiou and Moscicki, 2022; Sipos et al., 2021). Moreover, the microbiome has the potential to modulate immune competency in distant sites, thus impacting immune cell functionality and the release of cytokines (Chambers et al., 2020; Sipos et al., 2021). To fully comprehend these effects, it is necessary to investigate the intricate interactions between the microbiome, the immune system, and host tissues within relevant cancer models.

In addition to understanding the role of the endogenous microbiome in gynecologic cancer and its response to chemotherapy, it may be possible to apply exogenous microbes as treatment adjuvants. Precision therapy could leverage specific microbes or microbial functions for a targeted and personalized treatment. This method enhances efficacy by considering individual microbial variability affecting drug response. However, precise timing and dosing are essential to avoid disrupting microbial balance, which could cause infections, resistance, or immune reactions. Understanding the complex microbial ecosystem is key to preventing adverse effects and optimizing outcomes (Fernandes et al., 2022). Prospects for microbial-based cancer therapies could be developed using known metabolites from specific microbes. Understanding how microbial products influence immune function and cell signaling could lead to targeted cancer treatments. This framework could yield precise and safe therapeutic agents worthy of further exploration.

**Figure 1:**
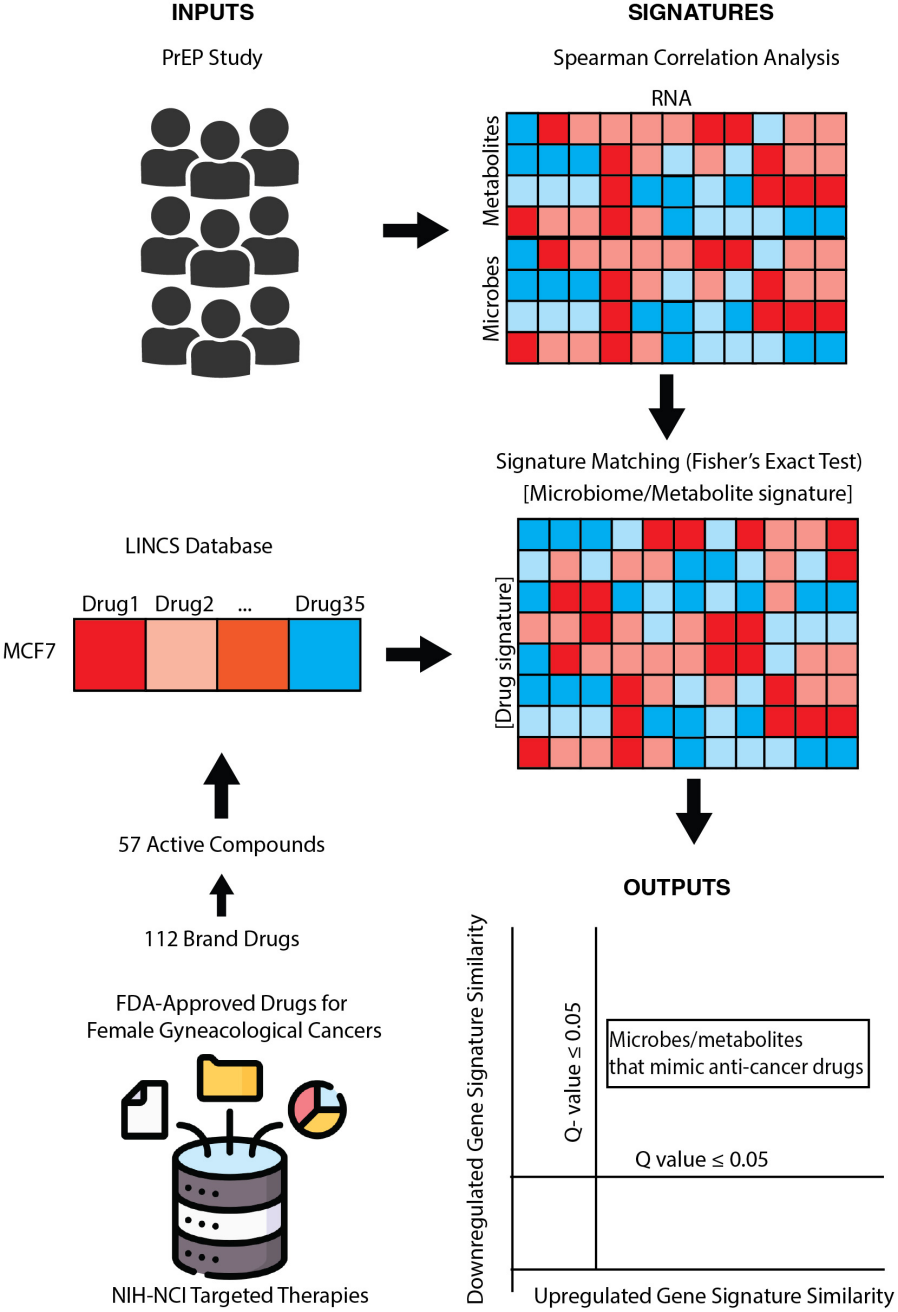
Pharmacobiome workflow: transcriptomics, metabolomics, and microbiome composition data from the partners PrEP vaginal microbiome multiomics datasets were analyzed via Spearman correlation analysis to extract microbe-host gene and metabolite-host gene signatures. National Institutes of Health-National Cancer Institute drugs screening in the LINCS Small Chemical Perturbation datasets were extracted to identify drug-host gene signatures. microbiome-gene and drug-gene up- and down-regulated signatures were compared via Fisher's exact test to identify anti-cancer drug: vaginal microbe and anti-cancer drug: vaginal metabolite signature similarities.

Here, we propose a systems biology computational framework to characterize the therapeutic potential of microbes and their products, termed the *Pharmacobiome*. The Pharmacobiome is the characterization of potential effects of specific microbiome communities and constituents on the host in terms of the known host-effects of drugs and pathways (Figure 1). The method principally functions as a computational high-throughput therapeutic screen of microbial taxa and microbiome-derived molecules where microbiome products that have a desired effect, through the lense of the effects of drugs, are selected based on predicted functional mimicry. Here, we apply this concept and methodology to identify new microbial products for precision treatment of cancers using microbial products in the *Anti-Gynecologic Cancer Pharmacobiome*. We address this objective by integrating vaginal microbiome multi-omics data from patients with and without bacterial vaginosis and *in vitro* drug transcriptomics data to identify vaginal microbes and microbial products that may have potential therapeutic applications for female reproductive cancers.

## Materials and methods

### Human vaginal microbiome multi-omics data

We obtained previously generated vaginal microbiome multi-omics data from a subgroup of 405 HIV-negative women enrolled in the Partners PrEP (Pre-Exposure Prophylaxis) study (Berard et al., 2023; Baeten et al., 2012). The transcriptomics, microbiome composition, and metabolomics data were previously generated (as described; Berard et al., 2023) from two sets of samples collected from participants with diverse exposures to HIV-risk factors (e.g., unprotected heterosexual sex, bacterial vaginosis, and other sexually transmitted infections). The groups in the original PrEP multi-omics study were largely concordant in their demographics and risk factors, where Group 1 included data from vaginal swabs collected from 315 participants aged 18–51 years and Group 2 included data from vaginal biopsies and swabs collected from 90 consenting participants (aged 25–51). Since the original study found minimal differences between the groups, we used the samples together for this present study.

For **transcriptomic analysis**, vaginal biopsies were collected from participants for RNA analysis, and were preserved in RNAlater solution to ensure integrity. These biopsies went through homogenization to disrupt tissue architecture, followed by RNA extraction using the RNeasy Fibrous Tissue Mini Kit (Qiagen), a method optimized for fibrous tissues to yield high-quality RNA. Samples underwent amplification with the Ovation PicoSL WTA System V2 and labeling with Encore BiotinIL kits before hybridization to HumanHT-12 v4 Expression BeadChips, a microarray platform specifically designed for genome-wide expression profiling. Expression data were generated in GenomeStudio (Illumina) software.

**Microbiome composition** was assessed through metaproteomics. Vaginal swabs were eluted with PBS, centrifuged, denatured, digested into peptides, and cleaned via reverse-phase chromatography. Peptides were quantified fluorometrically, and 1 μg per sample was analyzed by nano-LC-MS/MS on an LTQ Orbitrap Velos instrument. Proteins were identified by matching spectra against a curated TrEMBL database encompassing 16 bacterial genera, using Mascot v2.4.0 (Matrix Science) for peptide spectral matching. The protein identifications were validated in Scaffold ( ≤ 1% FDR) and genus-level abundance was derived from summed spectral counts of genus-specific proteins.

**Metabolomic profiling** involved extraction of metabolites from cervicovaginal lavages (CVL) using a 1:4 CVL:methanol ratio, followed by drying and resuspension in a buffer containing internal standards. To separate these metabolites, liquid chromatography-mass spectrometry (LC-MS) was performed using an Agilent 1200 HPLC system with ZIC-cHILIC column coupled to a Fusion Lumos Tribrid mass spectrometer. This system operated in both positive and negative ion modes, enhancing the detection of broad metabolite classes. For targeted analysis, 121 pre-selected metabolites associated with microbial and host pathways were quantified using parallel reaction monitoring (PRM), a high-resolution mass spectrometry mode that improves specificity. Raw spectral data were processed using Skyline, a software that integrates chromatographic peaks and aligns them with reference standards for precise quantification.

### Microbiome-associated host signature extraction

We analyzed transcriptomics, microbiome composition, and metabolomics data from Group 2 participants to investigate the relationships between microbial, metabolic, and host gene expression profiles. Using R Studio (Version 2022.12.0+353), we calculated Spearman correlation coefficients to assess microbe-RNA and metabolite-RNA associations. Standard Spearman correlation analysis was used to determine associations between microbial relative abundances and host transcriptomic profiles, effectively capturing monotonic relationships without assuming linearity. This approach suits the non-linear interactions between microbiota and host gene expression. For metabolome data, partial Spearman correlation analysis was applied, incorporating microbiome composition—specifically *Lactobacillus* dominance—as a covariate. This adjustment controlled for the confounding effects of dominant microbial taxa, such as *Lactobacillus iners* or *Lactobacillus crispatus*, allowing for a more explicit analysis of direct metabolite-host gene interactions. These methods reflect the intricate nature of host-microbiome-metabolome relationships and emphasize the need to consider biological context, such as dominant taxa, to improve the accuracy and clarity of the analysis.

Coded identifiers were used to match between datasets to ensure data integrity. Correlations were quantified using the Spearman coefficient (rho), which ranges from −1 to 1, indicating the strength and direction of associations, where positive values reflect upregulation and negative values indicate downregulation. We used the cor.test() function for correlation analysis and the p.adjust() function for multiple comparison corrections from the R stats package (version 4.3.0). This analysis identified significant correlations with adjusted *p*-values ≤ 0.05, generating lists of upregulated and downregulated genes influenced by 32 microbes and 99 metabolites.

### Identification of post-treatment drug transcriptomic perturbation data

The gene expression changes induced by 35 FDA-approved anti-cancer drugs were extracted from the Library of Integrated Network and Cellular Signatures (LINCS) Chemical Perturbation database (NCI, 2012; Todd Golub, 2021). Using the National Institutes of Health-National Cancer Institute database, we compiled a candidate list of 57 active compounds used as targeted therapies to treat various female gynecological cancers, including ovarian cancer, cervical cancer, vaginal cancer, vulvar cancer, and uterine cancer (NCI, 2012). In addition, we included therapies for breast cancer, recognizing that while breast cancer is not classified as a gynecologic malignancy, it serves as a prototypical model for hormone-responsive cancers. We compared the active compounds with the LINCS chemical perturbation transcriptomic database, identifying 35 critical (significant) drugs for further analysis (Todd Golub, 2021).

The LINCS Center for Transcriptomics catalogs gene expression profiles for cellular perturbagen molecules at different time points, doses, and cell lines using the L1000 assay. We obtained the LINCS data for the MCF7 breast cancer cell line because it serves as a prototypical hormone-responsive female cancer type for hypothesis generation and because it was the female cancer cell line with the most significant number of drugs screened at a 10 μM dose over 24 h, the most consistent criteria among the drugs analyzed.

### Comparison of drug gene signatures with microbiome host signatures

The microbes and metabolites signatures we obtained from Spearman correlations were compared to LINCS-derived signatures for similarities using a Fisher's Exact test which calculates exact *p*-value of the data and corrected for multiple hypothesis testing using the Benjamini Hochberg method on R software. Each microbe and metabolite gene list was compared to the gene list of each drug, where upregulated and downregulated genes were compared separately. Fisher's Exact Test was carried out using the fisher.test() function and the *p*-values correction using the p.adjust() function, both from the R stats package (version 4.3.0).

### Bacterial culture and sample preparation for metabolomics

*L. crispatus* ATCC 33820, *G. vaginalis* ATCC 14018, and *L. iners* ATCC 55195 were cultured for the metabolomics study. For *L. crispatus*, De Man–Rogosa–Sharpe media was used for suspension cultures (Reid, 1999). NYC III media was used for growing *G. vaginalis* suspension (Swidsinski et al., 2005). *L. iners* suspension culture was grown in BHI (Petrova et al., 2017). All cultures were grown at 37 °C, with 5% CO_2_ conditions for 24 h. Culture supernatants were collected after centrifuging at 5,000 g for 10 min followed by syringe filtration with a 0.22 μ filter to exclude all the bacterial cells. Samples were sent in triplicates to Metabolon, NC, USA, for non-targeted metabolomics (Jimenez et al., 2023). Metabolomics profiling was performed using the method described previously (Evans et al., 2009), and the data was stored in Metabolomics Workbench: http://dx.doi.org/10.21228/M82M8R.

### Cell viability and half-maximal inhibitory concentration (IC_50_)

The Cell Counting Kit-8 (CCK8) assay was carried out to determine cell viability and the IC_50_ of Taurine. Literature has shown Taurine and Cytosine to reduce cell viability at concentrations of 300 μM and above; hence, lower concentrations were tested in this study. Human endometrial stromal cells (HESC), Human endometrial cancer cells (HEC1A) and human breast cancer cells (MCF7) were grown to 80% confluency overnight at 37 °C and 5% CO_2_ on separate 96-well tissue culture plates in Dulbecco's modified Eagle's medium/F-12 Medium, McCoy's 5A Medium and Eagle's Minimum Essential Medium (EMEM), respectively. Then, Taurine was added at different concentrations (300, 100, 30, 10, 3, 1 μM), 20 μM Cisplatin (positive control), and DMSO (vehicle control) were added in triplicates and incubated for 24 h at 37 °C and 5% CO_2_. Next, 10 μL CCK8 solution was added to each well, and the plate was rocked gently to ensure even mixing. The cells were incubated at 37 °C and 5% CO2 for 4 h. The absorbance was determined using a BioTek Gen5 microplate reader and imager (Agilent Technologies, Santa Clara, CA) at 450 nm. A one-way analysis of variance (ANOVA) was performed to determine significance using Prism GraphPad Software (vs. 8.0, San Diego, CA, USA); *p*-values of ≤ 0.05 were considered significant.

### Data and code availability

Supplementary Tables have supplied all data, including Spearman coefficient calculation results, Fisher's exact calculation, gene lists, gene counts, and Cell viability assay results. Published data from Partners PrEP Study is available at GEO with accession number GSE139655 for Transcriptomics data and MetaboLights with accession number MTBLS7087 for Metabolomics data. LINCS Chemical Perturbation data is available at: https://maayanlab.cloud/sigcom-lincs/#/Download. The Bacterial Culture metabolomics data is available at: http://dx.doi.org/10.21228/M82M8R. All original code has been deposited at GitHub: https://github.com/Brubaker-Lab/Characterizing-the-Anti-Cancer-Potential-of-Vaginal-Microbes-and-Metabolites-.

## Results

### Integrative analysis of vaginal microbiome and host gene expression

In this study, we characterized the relationship between microbiome composition and RNA expression and the association between microbial metabolites and host vaginal epithelial RNA expression. We integrated metagenomic, metabolomic, and transcriptomic data from a well-characterized cohort of HIV-negative women in the Partners PrEP trial to comprehensively profile microbial communities, metabolite production, and host gene expression, enabling detailed analysis of host-microbe interactions relevant to gynecologic health and disease. For vaginal microbiota composition and taxa, we performed Spearman correlation analysis between 51 measured microbes and 23,482 gene expression signatures from vaginal biopsies. The size of the coefficient determined the effect magnitude, while the sign indicated the proposed regulatory direction on gene expression (Supplementary Tables S1, S2). Gene lists for each microbe were generated (adjusted *p* < 0.05), highlighting upregulated and downregulated genes.

To further explore connections between vaginal metabolites and host gene expression patterns, we integrated vaginal metabolomics and vaginal epithelial transcriptomics data from 23 matched participants. To generate metabolite-RNA correlations, we calculated partial Spearman correlation coefficients between 99 metabolites and 23,482 host genes controlling for *Lactobacillus* dominance across participants. Gene lists for each metabolite were generated (adjusted *p* < 0.05), highlighting upregulated and downregulated genes.

### Linking vaginal epithelial and microbiome gene signatures to anti-gynecologic cancer drug-gene signatures

Building upon the generated associations, we applied Fisher's exact test to compare gene expression profiles associated with vaginal microbes and metabolites to transcriptional changes induced by 35 chemotherapeutic agents, analyzing these relationships along two distinct dimensions (Supplementary Tables S3, S4). We defined “upregulated signature similarity” as the statistical concordance between genes positively correlated with microbial abundance and those upregulated by pharmaceutical compounds. Conversely, “downregulated signature similarity” captured the overlap between genes negatively associated with microbial presence and those suppressed by drug treatments. Drugs were organized into distinct clusters through hierarchical clustering analysis, employing complete linkage with Euclidean distance metrics to group compounds by their host-associated gene signature similarities. The resulting dendrogram reveals natural groupings that reflect underlying biological relationships, with closely related branches indicating drugs that induce similar cellular responses.

**Figure 2:**
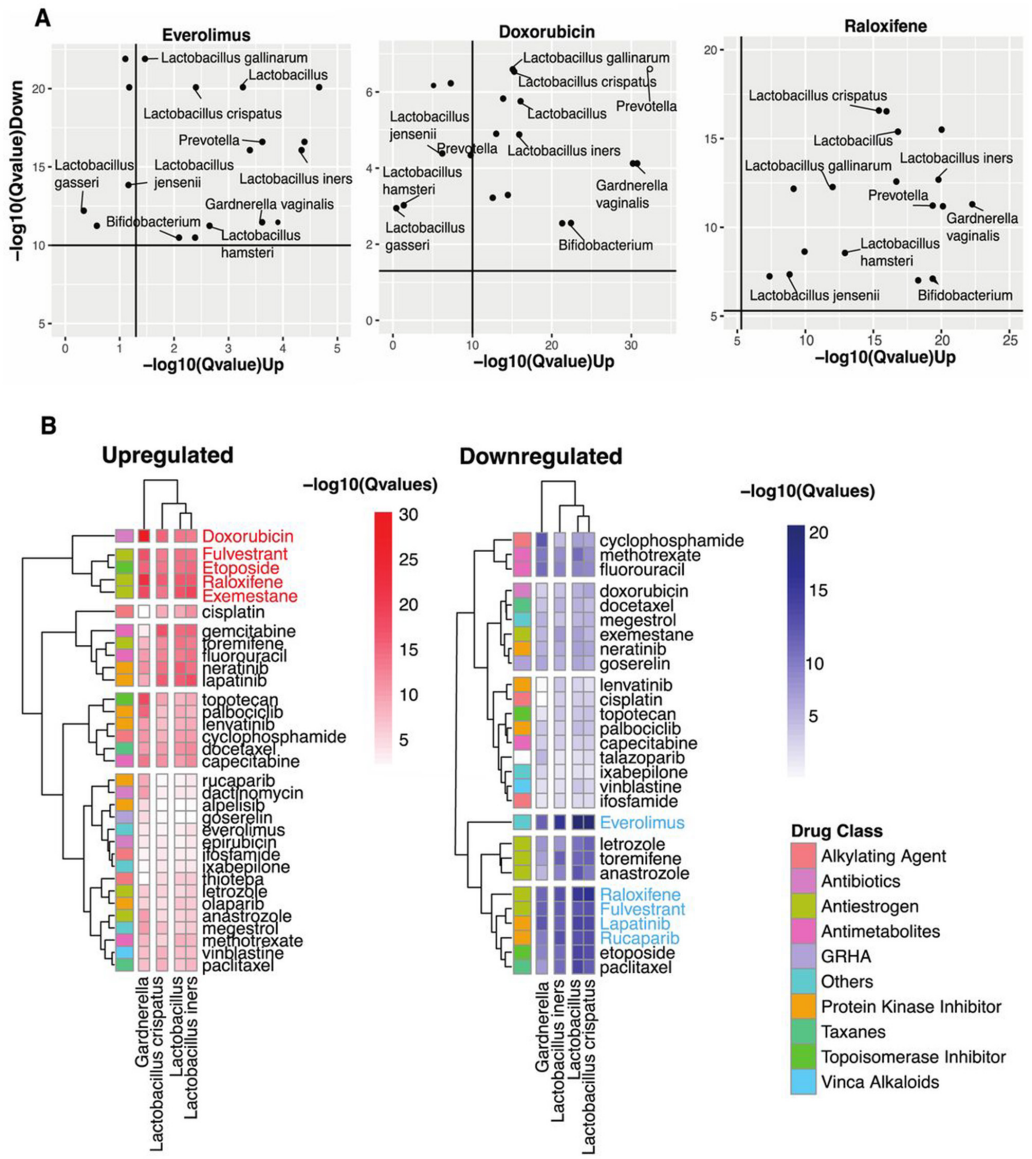
Identifying vaginal microbes with anti-cancer therapeutic potential. **(A)** Scatter plots of Everolimus, Doxorubicin, and Raloxifene show microbes with host-impacting gene signatures that significantly correspond to the drug-induced changes in host up-and-down-regulated genes. **(B)** Heatmap shows statistical concordance [–log_10_(*Q*-values)] between vaginal microbe-associated and drug-induced gene signatures for both upregulated and downregulated genes. Complete-linkage hierarchical clustering with Euclidean distance metrics reveals biologically relevant drug clusters based on host gene signature patterns. The drug Class key denotes the mechanism of action of the anti-cancer drugs. Note that mechanistically similar drugs cluster together, validating the computational approach internally.

**Figure 3:**
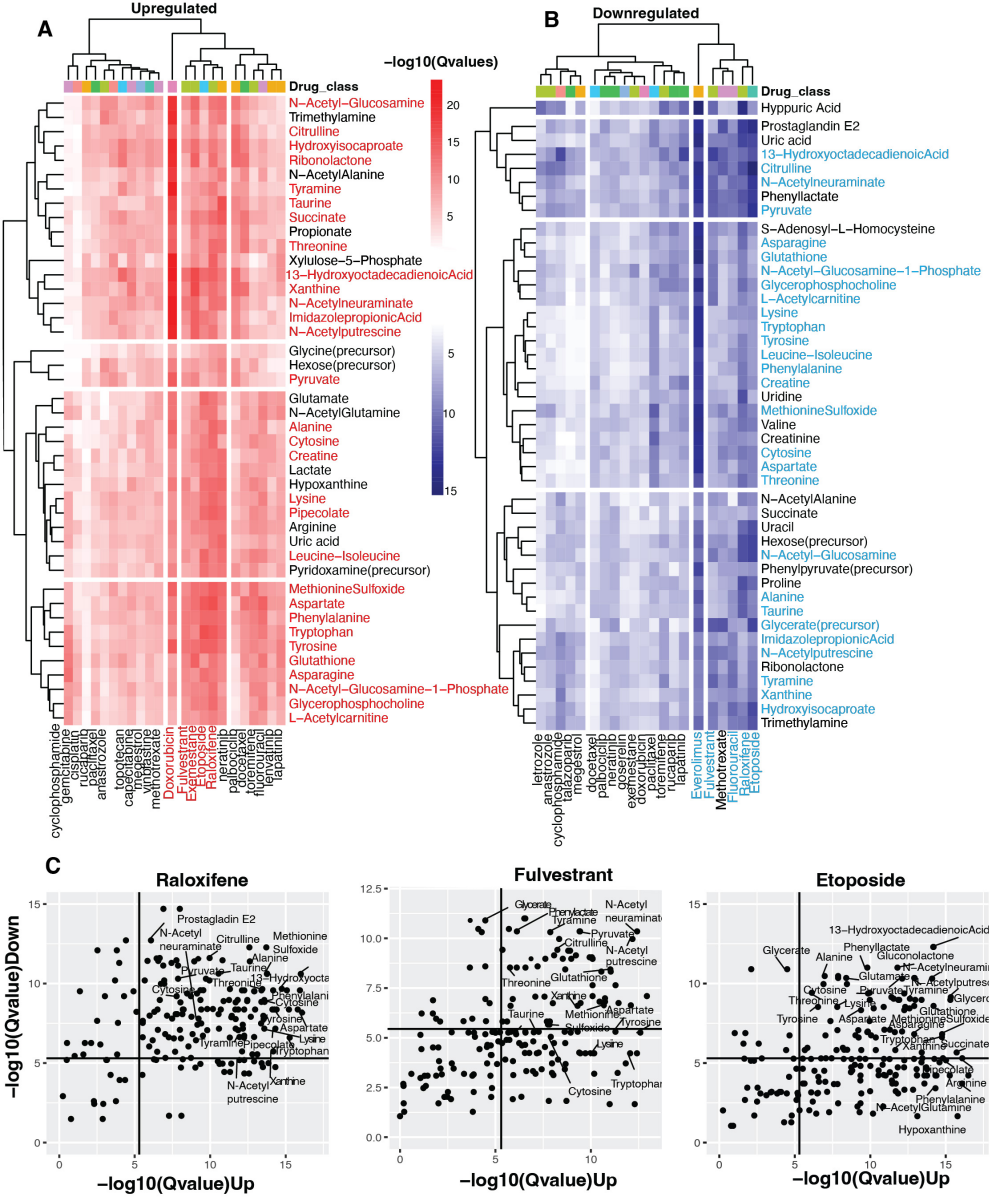
Identifying vaginal metabolites with anti-cancer potential. **(A, B)** Heatmap shows statistical concordance [–log_10_(*Q*-values)] between vaginal metabolite-associated and drug-induced gene signatures for both upregulated and downregulated genes. Complete-linkage hierarchical clustering with Euclidean distance metrics reveals biologically relevant drug clusters based on host gene signature patterns. The drug Class key denotes the mechanism of action of the anti-cancer drugs. Note that mechanistically similar drugs cluster together, validating the computational approach internally. **(C)** Scatter plots of Raloxifene, Fulvestrant, and Etoposide show metabolites with host-impacting gene signatures that significantly correspond to the drug-induced changes in host up-and down-regulated genes.

Notably, our unsupervised clustering approach revealed a striking pattern where drugs with established similar modes of action consistently clustered together. For example, steroidal aromatase inhibitors like Exemestane, selective estrogen receptor modulators (SERMs) like raloxifene, and selective estrogen receptor degraders (SERDs) like Fulvestrant formed a distinct cluster in the heatmap (Figures 2B, 3A). This pattern emerged naturally from the data rather than through any predetermined grouping, serving as an internal validation of our computational approach's ability to detect meaningful biological relationships.

### Identification of vaginal microbes with anti-cancer drug similarity based on gene signature alignment

Our analysis revealed striking patterns of functional mimicry. For upregulated signature similarity, five compounds demonstrated exceptional statistical significance: Doxorubicin, Fulvestrant, Etoposide, Raloxifene, and Exemestane. The downregulation axis identified a partially overlapping set of high-significance compounds: Everolimus, Raloxifene, Fulvestrant, Lapatinib, and Rucaparib. Figure 2A visualizes these transcriptional parallels between vaginal microbiota and three prominent anti-cancer drugs.

Everolimus, an mTOR inhibitor that suppresses lymphocyte activation by blocking growth-dependent signal transduction in T and B cells (National Institute of Diabetes Digestive Kidney Diseases, 2012), showed substantial downregulation similarity with *Lactobacillus crispatus* and other *Lactobacillus* spp. Doxorubicin, which disrupts DNA transcription through topoisomerase II inhibition (National Institute of Diabetes Digestive Kidney Diseases, 2012), demonstrated the most significant upregulation concordance with multiple vaginal microbes. Raloxifene, a selective estrogen receptor modulator that differentially activates or blocks estrogenic pathways in a tissue-specific manner (National Institute of Diabetes Digestive Kidney Diseases, 2012), exhibited substantial similarities in both regulatory directions. Across these three drugs, we observed significance-variable functional parallels with *Lactobacillus* spp., *Prevotella, Bifidobacterium*, and *Gardnerella* spp.

Narrowing our focus to four prevalent taxa found in over 70% of human vaginal samples (*Lactobacillus* genus, *L. iners, L. crispatus*, and *Gardnerella;* Figure 2B), we identified distinct patterns of drug-class similarity. *Lactobacillus* spp. demonstrated predominant functional overlap with antiestrogens and protein kinase inhibitors while showing substantial similarities with antimetabolites, topoisomerase inhibitors, antibiotics, and alkylating agents. However, *Gardnerella* shared some functional parallels with *Lactobacillus;* its strongest similarities clustered specifically within the protein kinase inhibitor class.

### Identification of vaginal metabolites with anti-cancer drug similarity based on gene signature alignment

Our analysis further revealed striking parallels between metabolite-gene associations and drug-induced transcriptional changes. Most notably, five compounds—Doxorubicin, Fulvestrant, Exemestane, Etoposide, and Raloxifene—demonstrated exceptional significance for upregulated gene similarity (Figure 3A). This indicates these pharmaceuticals activate transcriptional programs remarkably similar to those associated with certain vaginal metabolites. Conversely, Everolimus, Raloxifene, Fulvestrant, Etoposide, and Fluorouracil showed the strongest significance for downregulated gene signatures (Figure 3B), suggesting these agents suppress gene expression through pathways that mirror metabolite-mediated regulation.

The analysis identified over 40 vaginal metabolites with significant transcriptional parallels to 28 anti-cancer compounds (adjusted *p* < 0.05). Metabolite clusters associated with Fulvestrant, Raloxifene, and Etoposide—agents were particularly noteworthy, showing significant similarities in upregulation and downregulation signatures. These metabolite clusters comprised essential amino acids (threonine, phenylalanine, tryptophan, lysine, leucine-isoleucine), non-essential amino acids (alanine, methionine sulfoxide, taurine, asparagine, tyrosine, aspartate, citrulline), peptides (creatine, glutathione), sugars (N-acetylglucosamine, N-acetylneuraminate), and unsaturated fatty acids (13-hydroxyoctadecadienoic acid). Additional compounds included metabolites involved in amino acid and sugar metabolism (N-acetylglucosamine-1-phosphate, L-acetylcarnitine, hydroisocaproate, imidazole propionic acid, pipecolate), amines (N-acetylputrescine, tyramine), esters (glycerophosphocholine, glycerate), nucleic acids (xanthine, cytosine), and pyruvate (Figures 3A, B, Supplementary Table S5).

The three drugs showing the most consistent metabolite signature similarities—fulvestrant, raloxifene, and etoposide—represent distinct pharmacological classes (Figure 3C). Both raloxifene and fulvestrant target estrogen receptors, though through different mechanisms: raloxifene selectively modulates receptor activity, activating estrogenic pathways in some tissues while blocking them in others (National Institute of Diabetes Digestive Kidney Diseases, 2012), whereas fulvestrant creates pure antiestrogenic effects by inhibiting receptor dimerization and enhancing receptor degradation (Carlson, 2005). Etoposide operates through a different mechanism, forming complexes with DNA and topoisomerase II to hinder replication (Montecucco et al., 2015; Figure 3C).

### *Lactobacillus crispatus*-produced taurine inhibits the growth of endometrial cancer cells

To validate our computational findings regarding microbiome-derived anti-cancer metabolites, we conducted targeted metabolomic profiling of cultured vaginal bacteria and performed cell viability assays using both endometrial and breast cancer cell lines. Previous research has established *L. crispatus, L. iners*, and *G. vaginalis* as dominant members of the vaginal microbiota, each associated with distinct vaginal health states. *L. crispatus* dominance typically indicates a healthy vaginal environment, while *L. iners* predominance suggests a transitional, less stable condition. *G. vaginalis* dominance often correlates with dysbiotic states (Petrova et al., 2017; Jimenez et al., 2023; Castro et al., 2019).

**Figure 4:**
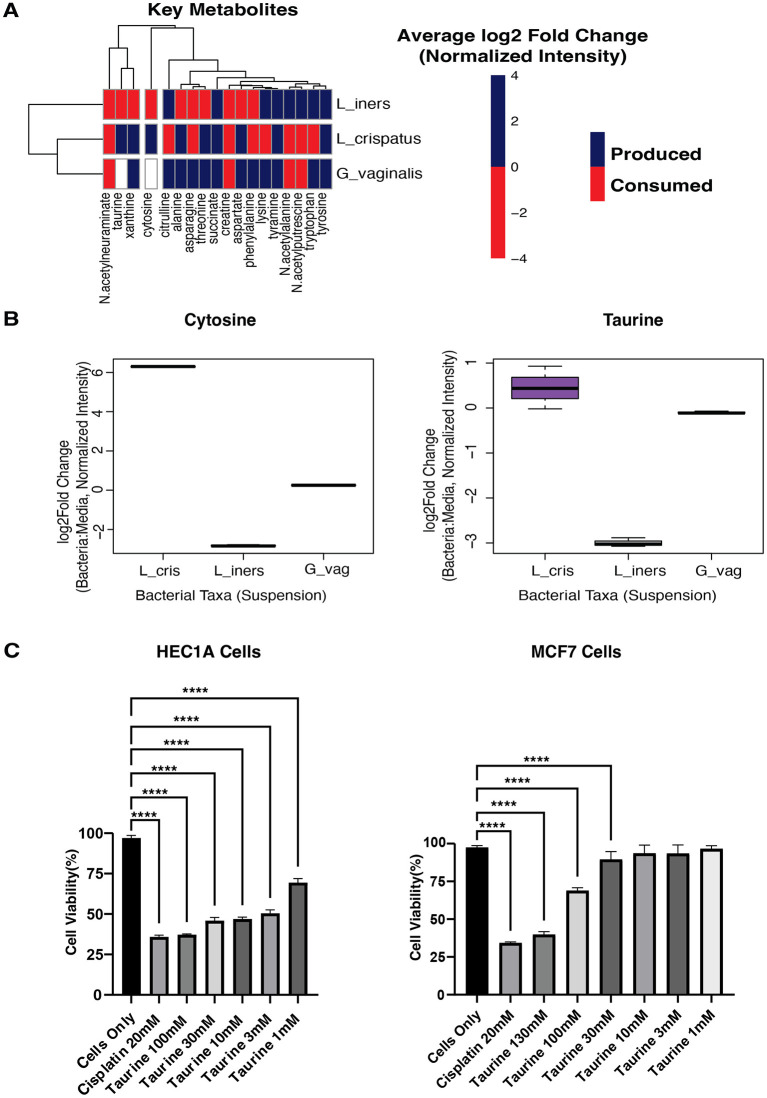
Metabolomics of cultured bacteria. **(A)** Heatmap showing the production/consumption of 18 Key Metabolites by L_crispatus, *Lactobacillus crispatus*; L_iners, *Lactobacillus iners*; G_vaginalis, *Gardnerella vaginalis*. **(B)** Boxplots showing the production of Cytosine and Taurine by L_cris, *Lactobacillus crispatus*; L_iners, *Lactobacillus iners*; G_vag, *Gardnerella vaginalis*. **(C)** Cell viability was determined using a CCK8 assay. Human endometrial cancer cells (HEC1A) and Human breast cancer cells (MCF7) were treated with Taurine at various concentrations of 100, 30, 10, 3, 1 μM for 24 h and spectrophotometrically measured at 450 nm. Cisplatin(positive control; chemotherapeutic drug) and all concentrations of Taurine significantly lowered the cell viability of HEC1A cells, while only the 100 and 300 μM concentrations of Taurine significantly lowered the cell viability of MCF7 cells when compared to Cells Only (no treatment). A one-way ANOVA was used for statistical analysis. A *p*-value of < 0.05 was considered significant. ^****^p < 0.0001. ^***^p < 0.001.

To explore which microbes were responsible for producing netabolites with anti-cancer potential, we cultured each microbe in suspension and analyzed their conditioned media using metabolomics. Our primary aim was to identify which vaginal microbes produce or consume the 28 metabolites with anti-cancer drug similarities identified in our previous analysis. Metabolic profiling revealed 597 metabolites produced or consumed across *L. crispatus, L. iners*, and *G. vaginalis*. Of particular interest, 18 key metabolites with anti-cancer potential were detected in bacterial culture media, confirming their microbial origin (Figure 4A). Of these, *L. crispatus* uniquely produced Taurine and Cytosine, while *L. iners* demonstrated active consumption of these same compounds. In contrast, *G. vaginalis* neither produced nor consumed them (Figure 4B). These findings suggest a specialized metabolic role for L. crispatus in producing compounds with potential anti-cancer properties. Conversely, *L. iners'* consumption pattern may effectively reduce the bioavailability of these protective metabolites, potentially diminishing their cancer-inhibitory effects in the vaginal environment.

To test direct anti-cancer activity of these metabolites, we evaluated taurine and cytosine in cell viability assays using MCF7 cells (the breast cancer line used for LINCS database gene signature generation), HES cells (endometrial stromal cell line), and HEC1A cells (an endometrial cancer line). We selected taurine and cytosine for further investigation based on two compelling factors: our metabolomic findings and existing literature. Previous research has demonstrated taurine's anti-cancer properties in breast cancer models (Zhang et al., 2015; Shennan and Thomson, 2007), and its significant role in estrogen metabolism and detoxification (Li et al., 2022). Similarly, cytosine derivatives have shown promise in various cancer contexts (Kim et al., 2024; Ligasová et al., 2023; Christensen et al., 2010). Despite this evidence, neither compound has been adequately evaluated in gynecologic malignancies, presenting a vital knowledge gap given their *L. crispatus*-specific production in the vaginal microenvironment and their transcriptional similarities to established anti-cancer drugs.

We treated all cell lines with taurine and cytosine at concentrations ranging from 1 to 300 μM for 24 h, using Cisplatin as a positive control. Cell viability was quantified using the CCK8 assay. Taurine demonstrated remarkable tissue and malignancy selectivity in our cellular assays. While having no detectable effect on normal HES endometrial stromal cells (Supplementary Table S6), taurine significantly inhibited HEC1A endometrial cancer cell viability at all concentrations tested (*p* < 0.001). MCF7 breast cancer cells showed intermediate sensitivity, responding only to higher taurine concentrations (30, 100, and 130 μM; Figure 4C). These findings reveal that taurine, produced explicitly by *L. crispatus* in the vaginal microenvironment, exhibits selective toxicity toward endometrial cancer cells while sparing normal endometrial tissue and showing reduced activity against distant malignancies. This tissue-specific anti-cancer profile suggests taurine may represent a microbiome-derived compound relevant to gynecologic cancer prevention. In contrast, cytosine demonstrated no effect on the cell lines at the concentrations tested (Supplementary Table S6).

## Discussion

We developed the Pharmacobiome as a novel systems biology approach to characterize microbes and microbial products' function relative to known drugs. This methodology enables systematic comparison between microbial effects and established pharmaceuticals, opening new avenues for therapeutic discovery. To explore this concept, we integrated vaginal microbiome multi-omics data from HIV-negative study participants with *in vitro* drug screening transcriptomics to identify microbes and microbial products with potential efficacy against female reproductive cancers.

Our analysis revealed *Lactobacillus* spp. (including *L. iners* and *L. crispatus*) and *Gardnerella* as predominant microbes, detected in over 70% of patient samples. This composition aligns with established research by Ravel et al. that classified vaginal microbiota into five major community state types (CSTs): four dominated by *Lactobacillus* spp.; *Lactobacillus crispatus, Lactobacillus gasseri, Lactobacillus iners*, and *Lactobacillus jensenii* (CST-I, II, III, and V), while the fifth is non-*Lactobacillus* dominant but instead dominated by polymicrobial obligate anaerobes like *Gardnerella* (CST-IV; Ravel et al., 2011).

Understanding the natural diversity of vaginal microbiota is crucial for interpreting our findings. Research has demonstrated that *Gardnerella vaginalis*, often associated with bacterial vaginosis, can exist as part of a healthy vaginal microbiome. Multiple studies have found *G. vaginalis* in 25–38.5% of women with normal Nugent scores and predominant lactobacilli flora (Hickey and Forney, 2014). One particular study detected *G. vaginalis* in 36% of women, with only a subset experiencing symptoms (Himschoot et al., 2025). The (Cleveland Clinic, 2021) has confirmed that *G. vaginalis* naturally coexists with other bacteria in balanced vaginal flora and only causes issues when overgrowth occurs (Cleveland Clinic, 2021). Furthermore, research suggests strain variations are significant, with some *G. vaginalis* strains being non-pathogenic in healthy women (Abou Chacra et al., 2022). This evidence challenges the traditional views that *G. vaginalis* is inherently pathogenic, suggesting instead that bacterial balance rather than mere presence determines vaginal health (Hickey and Forney, 2014; Cleveland Clinic, 2021).

Our comprehensive analysis identified 28 metabolites, and three microbes, *L. crispatus, L. iners*, and *Gardnerell*a, were significantly similar in their host-associated gene signatures to host gene signatures to those induced by various anti-cancer drugs. We performed untargeted metabolomics on cultured *L. crispatus, L. iners*, and *G. vaginalis to determine which microbes produce anti-cancer metabolites*. The focus on *L. crispatus* was particularly significant as this spp. is generally associated with optimal, beneficial vaginal microbiota. Understanding the metabolites it produces provides critical insights into how this microbe might contribute to cancer protection. Conversely, we recognized that consuming metabolites with anti-cancer potential by *G. vaginalis* or other bacteria might hinder cancer treatments' effectiveness. These interactions highlight the complexity of the vaginal microbiome environment and emphasize the importance of monitoring bacterial interactions in the context of cancer treatment. Understanding these dynamics is essential to ensure optimal patient outcomes and potentially develop microbiome-based therapeutic approaches.

The convergence of our computational and experimental bacterial metabolomics analysis pointed toward *L. crispatus-produced* Taurine as a potential vaginal microbiome-derived anti-cancer agent. Taurine, an essential intracellular amino acid in humans (Santulli et al., 2023; Ripps and Shen, 2012), has well-documented antioxidant and anti-inflammatory properties (Jong et al., 2021; Baliou et al., 2021). However, research examining its impact on tumors has been limited, and potential antitumor mechanisms remain largely unexplored. To test our hypothesis, the anti-cancer activity of Taurine was assessed by treating HES endometrial stromal, HEC1A endometrial cancer, and MCF7 breast cancer cell lines with varying concentrations of Taurine for 24h. Taurine concentrations exhibit remarkable tissue-specific variation throughout the human body, with exceptionally high levels in excitable tissues. Within the retina's photoreceptor cells, taurine reaches concentrations as high as 70 mM, representing one of the highest cellular concentrations found in human tissues (Seidel et al., 2019).

In contrast, circulating taurine levels are substantially lower, with concentrations ranging from 7 to 200 μM in plasma and whole blood, variations that depend on an individual's feeding state and metabolic condition (Santulli et al., 2023). Our experiments explored physiological concentrations, and the results revealed that at concentrations ranging from 130 μM to as low as 1 μM, taurine significantly reduced the viability of HEC1A endometrial cancer cells. Meanwhile, MCF7 breast cancer cells only showed a significant reduction in cell viability at concentrations of 100 μM and above. This differential response indicates that taurine's cytotoxic potential is cell-type dependent, suggesting specificity in its anti-cancer effects.

While taurine is essential in various physiological functions like redox balance and cell volume regulation, our findings suggest that elevated concentrations may disrupt cell metabolism and growth, triggering protective responses or promoting cell death (Centeno et al., 2024). This dual nature of taurine makes it beneficial at physiological levels but potentially cytotoxic to specific cancer cells at higher concentrations, presenting an intriguing therapeutic opportunity. Further studies may need to be conducted to determine if Taurine induces apoptosis in other gynecological cancers and in primary non-cancerous cells to understand the molecular mechanism and differences in cytotoxicity in detail.

Our analysis further reveals that *L. crispatus* induces host gene signatures similar to established chemotherapeutic drugs, specifically Raloxifene, Fulvestrant, Exemestane, and Etoposide. Each drug targets cancer through distinct mechanisms, providing insights into how *L. crispatus* might confer protective effects.

Raloxifen acts as a selective estrogen receptor modulator (SERM) that differentially activates or blocks estrogenic pathways in a tissue-specific manner. Breast tissue binds to estrogen receptors competitively, preventing estrogen from stimulating cancer cell growth while preserving beneficial estrogenic effects in bone tissue (National Institute of Diabetes Digestive Kidney Diseases, 2012).

Fulvestrant functions as a selective estrogen receptor degrader (SERD) that binds to estrogen receptors in cancer cells, causing their destabilization and subsequent degradation. This process ultimately blocks estrogen's ability to promote cancer cell proliferation by eliminating the receptors themselves rather than simply blocking them (Nathan and Schmid, 2017).

Exemestane belongs to the aromatase inhibitor class, working by irreversibly binding to and inactivating the aromatase enzyme. This enzyme is essential for converting androgens into estrogen, and its inhibition reduces estrogen levels in peripheral tissues, thereby slowing the growth of estrogen-dependent breast cancer cells (Buzdar, 2000).

Etoposide works through dual mechanisms, combining direct cytotoxicity with immune system activation. It forms complexes with topoisomerase II and DNA, causing unrepairable double-stranded breaks that prevent mitosis and trigger cell death (Zhang et al., 2021). Beyond this primary action, etoposide activates the innate immune response through the STING pathway via IFI16, ATM, and PARP-1 proteins (Dunphy et al., 2018). It selectively eliminates pathologically activated T cells while preserving quiescent T cells, making it effective against hyperinflammatory conditions (Johnson et al., 2014). In cancer therapy, etoposide enhances MHC class I expression on cancer cells, increasing their visibility to cytotoxic T cells while generating neoantigens that stimulate antitumor immune responses (Mouw et al., 2017). This dual mechanism expands etoposide's therapeutic profile beyond simple DNA damage.

The gene expression similarities between *L. crispatus* and these hormone-modulating drugs (Raloxifene, Fulvestrant, and Exemestane) are particularly significant when considering the vaginal microbiome's relationship with hormonal regulation. Several studies have shown that estrogen stimulates glycogen deposition in vaginal epithelial cells (Mirmonsef et al., 2015; Li et al., 2018), creating an optimal environment for beneficial bacteria. This glycogen is a crucial substrate for lactobacilli spp., which ferments it into lactic acid (O'Hanlon et al., 2013). The resulting acidic microenvironment (pH 3.5–4.5) creates a natural defense mechanism that inhibits pathogen growth while maintaining vaginal homeostasis (O'Hanlon et al., 2013, 2019). What's remarkable is how this process self-regulates; lactobacilli naturally cease their metabolic activity once optimal acidity is reached (O'Hanlon et al., 2013). This acidic environment strengthens the epithelial barrier (Li et al., 2018), significantly reducing susceptibility to infections (O'Hanlon et al., 2013). Statistical analysis confirms the biological significance of this relationship, with studies showing an exceptionally strong correlation between vaginal pH and lactobacilli dominance (*r*^2^ = 0.91–0.97). While progesterone may inversely affect soluble glycogen levels (Mirmonsef et al., 2015, 2016), an estrogen-driven proliferation of epithelial cells ensures sustained glycogen availability for lactic acid synthesis (Li et al., 2018). This hormonal balance maintains the protective ecosystem that makes *L. crispatus* and related spp. important components of vaginal health.

Beyond maintaining vaginal acidity, *Lactobacillus* spp. protects against cancer through complex interactions with the immune system. *Lactobacillus reuteri*, for instance, secretes compounds that promote dendritic cell maturation (marked by increased CD83 and CD86 expression) while triggering anti-inflammatory IL-10 production that helps maintain immune balance (Engevik et al., 2021). Lactobacilli influence extends to T cells as well; *Lactobacillus rhamnosus Lcr35* increases regulatory T cells (CD4^+^CD25^+^Foxp3^+^ T_regs_) that prevent excessive inflammation, while promoting TLR-2-dependent production of transforming growth factor-β, IL-10, and IL-8 (Ding et al., 2017).

Recent research has identified specific anti-cancer metabolites produced by lactobacilli. *Lactobacillus gallinarum* generates indole-3-carboxylic acid, which blocks IDO1 expression and improves CD8^+^ T cell function. Additionally, *Lactobacillus-*produced acetate increases interferon-γ expression and promotes CD8^+^ T lymphocyte infiltration into tumors, slowing cancer growth (Fong et al., 2023). These findings suggest that targeted microbiome modulation focusing on *Lactobacillus* may offer promising new approaches to cancer prevention.

Our findings suggest new possibilities for utilizing *Lactobacillus crispatus* or its products in cancer therapy. Vaginal microbiota-derived molecules present several significant advantages over conventional chemotherapeutic approaches for gynecologic cancers. Unlike broad-spectrum chemotherapy agents that often damage healthy cells, microbiome-derived molecules may exert more targeted effects, selectively influencing specific mechanisms involved in cancer development or progression (Lin et al., 2024). Additionally, since these molecules originate from the host's microbiome, they are likely to be better tolerated with fewer side effects than traditional chemotherapy drugs. The role of the vaginal microbiome in immune function further suggests that these molecules may have the capacity to stimulate the immune system to combat cancer cells—adding another dimension to their therapeutic potential (Rizzo et al., 2021; Xia et al., 2023).

Despite the promising findings presented here, it is crucial to acknowledge that this field of research is still in its infancy. Further investigations are necessary to ascertain the efficacy and safety profiles of these vaginal microbiome-derived molecules for gynecologic cancer treatment (Han et al., 2023). Nevertheless, the distinctive advantages offered by these natural compounds, including potentially fewer side effects and more targeted action, strengthen their considerable therapeutic potential.

In conclusion, our study shows that commensal microbes like *Lactobacillus crispatu*s and metabolites like Taurine can induce cytotoxic effects on cancer cells comparable to chemotherapeutic drugs. The Pharmacobiome analytical framework we have developed establishes a quantifiable relationship between microbial communities and therapeutic outcomes, creating numerous pathways for innovative cancer treatment strategies.

The applications of microbial products for cancer therapy extend far beyond the specific bacteria and metabolites discussed in this study. The field has massive potential for future growth and innovation. Our Pharmacobiome analysis provides a robust framework for discovering novel therapeutic strategies potentially applicable to gynecologic cancers and other conditions. This approach is based on profiling the therapeutic actions of human microbiome communities. By understanding and harnessing the natural anti-cancer properties of the vaginal microbiome, we may develop more targeted, better-tolerated therapies that work with the body's natural defenses rather than against them. Our results indicate that specific components of the vaginal microbiome have the potential to modulate host responses that are relevant to cancer therapy. The observation highlights the need for additional experimental and translational studies to fully assess the potential for clinical application.

## Future directions

While our study provides foundational insights into the therapeutic potential of *L. crispatus*-derived metabolites like taurine, yet several critical questions remain to bridge these findings to clinical translation. Although our *in vitro* assays demonstrated that taurine selectively reduces the viability of endometrial cancer cells, the precise mechanisms responsible for this effect remain unclear. It has not yet been determined whether taurine induces apoptosis, alters cell cycle progression, or modulates specific immune pathways. To address this gap, future studies will focus on mechanistic investigations using annexin V and propidium iodide staining to assess apoptosis, flow cytometry-based analyses to monitor changes in the cell cycle, and co-culture systems that incorporate key immune cell populations. Collectively these approaches will provide insight into how taurine and other microbiome metabolites influence cancer cell fate.

Moving forward, our research will focus on two major areas to support clinical translation. First, a comprehensive pharmacokinetic and systemic safety profiling is essential. Although, our *in vitro* assays showed taurine's selective cytotoxicity against endometrial cancer cells (HEC1A), its *in vivo* absorption, distribution, metabolism, and excretion (ADME) properties require thorough investigation. Physiological taurine concentrations range from 7 to 200 μM in plasma to 70 mM in retinal tissues, necessitating careful dose optimization studies in animal models to determine therapeutic windows. We propose intravaginal hydrogel delivery systems to achieve localized, high-concentration taurine exposure (>1 mM), minimizing systemic absorption and off-target effects. Concurrently, toxicology studies in murine models will evaluate metabolite safety at tissue-relevant doses, addressing potential off-target effects.

Then, we will develop translational models that recapitulate host-microbe interactions and capable of replicating the complexity of human tissue-microbiome crosstalk. To address this, we propose three-dimensional vaginal organoids co-cultured with *L. crispatus* biofilms under hypoxic conditions (5% O_2_) to model metabolite gradients and epithelial-stromal signaling. Orthotopic endometrial cancer models using humanized mice with vaginal microbiota transplants (*L. crispatus* vs. *L. iners*) to test taurine's tumor-suppressive effects *in vivo*. Finally, *ex vivo* patient-derived cervical and endometrial tissue explants exposed to taurine, enabling real-time tracking of immune cell recruitment (e.g., M1/M2 macrophages) via spatial transcriptomics.

By integrating these approaches, we aim to advance microbiome-derived metabolites from bench to bedside, ensuring efficacy and safety in the complex landscape of human physiology.

## Data Availability

The Supplementary Tables include all raw data, encompassing Spearman coefficient calculation results, Fisher's exact test outcomes, gene lists, gene counts, and cell viability assay results. Published data from the Partners PrEP Study can be accessed at the GEO repository (accession number GSE139655) for transcriptomics data and MetaboLights (accession number MTBLS7087) for metabolomics data. LINCS chemical perturbation data is available through Ma'ayan Lab's SigCom LINCS. Bacterial culture metabolomics data can be found at doi: 10.21228/M82M8R. All original code used in this study has been deposited at https://github.com/Brubaker-Lab/Characterizing-the-Anti-Cancer-Potential-of-Vaginal-Microbes-and-Metabolites-.
